# Tale of Two Fellows

**DOI:** 10.1200/JGO.17.00050

**Published:** 2017-08-21

**Authors:** Karin Purshouse, Twalib Ngoma, David Kerr

**Affiliations:** **Karin Purshouse** and **David Kerr**, University of Oxford, Oxford, United Kingdom; and **Twalib Ngoma**, Muhimbili University of Health and Allied Sciences, Dar es Salaam, Tanzania.

The Scots have a saying, “When friends meet, hearts warm.” And such has been the friendship between Twalib Ngoma and David Kerr since they met as Fellows at Glasgow’s Beatson Cancer Institute in the 1980s, where Twalib showed data suggesting that the majority (80%) of Tanzanians presented with late stage 4 cancer. In conversation in Dar es Salaam some 30 years later, despite the significant downward stage shifts and improvements in cancer survival seen in Europe and the United States, it was apparent that there have been no such steps forward in most sub-Saharan African nations. It remains true that although the continent of Africa as a whole has a relatively low overall incidence rate of cancer, patients in less-developed countries also unfortunately have the poorest prognosis with the highest numbers of deaths. Much of this can be attributed to late-stage diagnosis.

These sorts of anecdotes prompted *Journal of Global Oncology* to conduct a survey of ASCO members who practiced in low- and middle-income countries (LMICs) to ask them to list the factors that hindered their ability to deliver high-quality cancer care to their citizens. Apart from the generic complaint of lack of resources (financial, human, and infrastructural), late presentation of disease and tumor burden were cited as the greatest barrier to improving the outcome for their patients. Tumor volume and stage correlate with prognosis for a variety of reasons—metastasis leading to multiorgan failure, reduction in performance status and cachexia, and increased tumor heterogeneity, with an associated increase in the likelihood of anticancer drug resistance. In short, the opportunity to diagnose, treat, and potentially cure cancer early in LMICs is being lost.

The benefits of screening for cervical, breast, and colorectal cancer in the West have been demonstrated, but the costs of establishing and maintaining conventional screening infrastructure is high and places these interventions at the margins of cost effectiveness, even in wealthy countries where cancer incidence is relatively high. Given this, we wanted to challenge the wider oncology community that, rather than adopt a one-size-fits-all approach to earlier disease detection or downstaging on the basis of Western screening models, was it possible to identify, develop, and deliver simpler, effective methods that could be used in prospective trials to demonstrate a population shift in tumor burden at presentation?

Some have proposed turning the problem on its head: rather than getting patients to come to a medical center for screening, take screening to them. Training female village-based volunteers to perform a clinical breast examination was just one pilot intervention that demonstrated potential in increasing awareness and pick-up rate of early-stage breast cancer.^1^ Cervical cancer rates have declined significantly since the introduction of cervical smear screening programs, but the logistic and cultural challenges surrounding this also required an alternative approach. Using the Visual Inspection with Acetic Acid test, whereby a see-and-treat approach is used to treat abnormal areas, has resulted in a notable reduction in cervical cancer incidence.^2^ More technological advances include the development of a microendoscope with a build-it-yourself computer and dye kit, which allows the cervix to be visualized and assessed in the same sitting.^3^ Geography is a key problem that any downstaging program must overcome. In Tanzania, there is only one tertiary cancer center (the Ocean Road Cancer Institute) to manage the estimated 20,000 new diagnoses of all cancers nationwide each year. This means screening for cancer must be feasible in smaller hospitals and medical centers across the country and must be acceptable to the population it is serving. Screening acceptability is just one of the issues identified by the African Research Group for Oncology (ARGO), a collective that aims to learn from the development and maintenance of a cancer research consortium in Nigeria.^4^ The key messages are of different population expectations, a different oncology management structure that is surgically centered, and the challenges of accessing, say, stool testing and colonoscopy services if colorectal cancer screening was to be implemented, although this is a controversial theme in LMICs.^5^

To this end, we have commissioned a special issue of *Journal of Global Oncology* as a call to arms: we must identify novel screening tools that are cheap, immediate, and effective. Existing screening tools cannot be relied on to address this pressing need in LMICs with the logistic, financial, and cultural limitations they present. Diagnosing cancer early is vital to improve patient outcomes and health care efficiencies, particularly with the significant predicted increase in cancer incidence on the horizon.^6^ We hope that we can stimulate a debate and action beyond the conventional readership of our journal to engage not only physicians, front-line health workers, and public health specialists but also social and technology entrepreneurs and existing high-tech companies to consider this problem and, from this, research laterally useful methodologies. Let us invent a hand-held, solar-powered ultrasound machine that is digitally interpretable and could be used by two old friends: Kerr and Ngoma.
